# Characterization and Fitness Cost of Tn*7100*, a Novel Integrative and Conjugative Element Conferring Multidrug Resistance in *Haemophilus influenzae*

**DOI:** 10.3389/fmicb.2022.945411

**Published:** 2022-07-22

**Authors:** Helene Johannessen, Inger Lill Anthonisen, Nermin Zecic, Kristin Hegstad, Trond Egil Ranheim, Dagfinn Skaare

**Affiliations:** ^1^Department of Microbiology, Vestfold Hospital Trust, Tønsberg, Norway; ^2^Norwegian National Advisory Unit on Detection of Antimicrobial Resistance, Department of Microbiology and Infection Control, University Hospital of North Norway, Tromsø, Norway; ^3^Research Group for Host-Microbe Interactions, Department of Medical Biology, Faculty of Health Sciences, UiT the Arctic University of Norway, Tromsø, Norway; ^4^Department of Microbiology, Fürst Medical Laboratory, Oslo, Norway

**Keywords:** integrative and conjugative element, ICE, multidrug resistance, MDR, *Haemophilus influenzae*, fitness, conjugation, transposon

## Abstract

A multidrug-resistant (MDR) strain of *Haemophilus influenzae*, Hi-228, with phenotypic resistance toward ampicillin, cefotaxime, chloramphenicol, gentamicin, and azithromycin, was isolated in Oslo, Norway. The strain was part of a clonal outbreak (2016–2017) comprising five ST143 strains with identical resistotypes. Hi-228 carries a novel integrative and conjugative element (ICE), Tn*7100*, contributing to this remarkable and previously unreported MDR profile. Tn*7100* contains the following resistance genes: *bla*_TEM−1B_, *catA2, aac(6*′*)*-Im, *aph(2*″*)*-Ib, *mef* (E), and *mel*. The latter four are previously unreported or rarely reported in *H. influenzae*. In this study, we investigated the genetic environment, mechanisms of transfer, impact on phenotypic susceptibility, and fitness cost of this ICE. We found that Tn*7100* has an overall structure similar to the previously described ICE Tn*6686*, with *bla*_TEM−1B_ and *catA2* carried by Tn*3* and Tn*10*, respectively. The major difference between Tn*7100* and Tn*6686* is that Tn*7100* lacks *tet*(B) but carries the resistance gene pairs *aac(6*′*)*-Im and *aph(2*″*)*-Ib and *mef* (E) and *mel*. The gene pairs are located on the novel transposable elements Tn*7470* and Tn*7471*, which have high sequence identities to a plasmid in *Enterobacterales* and an ICE in streptococcal species, respectively. Tn*7100* does circularize and is transferable, however, at a low frequency. Head-to-head competition experiments showed that uptake of Tn*7100* reduces bacterial fitness. Our study shows that MDR strains are capable of clonal spread and that the *H. influenzae* supragenome comprises an increasingly wide range of transferable resistance genes, with evidence of transfer from unrelated genera. The findings offer a glimpse into the genome dynamics of *H. influenzae*, highlighting the importance of rational antibiotic usage to contain antimicrobial resistance and the emergence of MDR strains in this important pathogen.

## Introduction

*Haemophilus influenzae* is a Gram-negative bacterium colonizing the respiratory tract in humans and causing both invasive and non-invasive infections. Non-encapsulated (non-typeable) *H. influenzae* (NTHi) frequently causes eye, ear, sinus, and respiratory tract infections, including complicating infections in cystic fibrosis and exacerbations in chronic obstructive pulmonary disease (COPD). In fact, *H. influenzae* is one of the most common causes of exacerbations in COPD. *H. influenzae* also has the potential to cause septic pneumonia and meningitis, with a particularly high risk in infants, immunocompromised, and elderly people (Van Eldere et al., 2014). Several reports suggest that the incidence of invasive NTHi disease in the general population is increasing, and Norway and Sweden have the highest incidence rates in Europe (ECDC, 2019).

The emergence and spread of beta-lactam-resistant strains with transferable beta-lactamases and/or alterations in penicillin-binding protein 3 (PBP3) has eliminated aminopenicillins and threatens extended-spectrum cephalosporins, as safe empiric therapeutic options in severe *H. influenzae* infections (Van Eldere et al., 2014). Accordingly, ampicillin-resistant *H. influenzae* was added to the WHO priority pathogen list for research and development of new and effective antibiotics in 2017 (WHO, 2017). The situation is further complicated by the dissemination of multidrug-resistant (MDR) *H. influenzae* with acquired resistance to the most suitable non-beta-lactam agents such as trimethoprim-sulfamethoxazole, tetracyclines, quinolones, chloramphenicol, and/or macrolides (Pfeifer et al., 2013; Kuo et al., 2014; Skaare et al., 2014; Seyama et al., 2017; Hegstad et al., 2020).

Integrative and conjugative elements (ICEs) are self-transmissible mobile genetic elements ranging from ~20 to >500 kb in size. They primarily reside in the chromosome of one bacteria but can excise, circularize, and transfer to another bacteria (Wozniak and Waldor, 2010; Johnson and Grossman, 2015). Horizontal gene transfer by ICEs is an important mechanism for the dissemination of antibiotic resistance between bacteria, and ICEs may carry a wide range of resistance genes mediating resistance to most types of antibiotics (Juhas et al., 2007b; Li et al., 2016; Partridge et al., 2018; Botelho and Schulenburg, 2020; Hegstad et al., 2020; Nikolaou et al., 2020). ICE*Hin1056* has promoted bacterial diversity and adaptation in *H. influenzae* by becoming an efficient vector of antibiotic resistance (Mohd-Zain et al., 2004; Juhas et al., 2007b). Conjugation of this ICE is enabled by the type IV secretion system (T4SS), and ICE*Hin1056*, as well as other ICEs, integrate site-specifically into the tRNA^Leu^ gene in *H. influenzae* (Dimopoulou et al., 2002; Juhas et al., 2007a; Hegstad et al., 2020).

Several novel ICEs conferring MDR phenotypes were recently described in *H. influenzae* (Hegstad et al., 2020), underlining the ability of this organism to acquire a variety of antibiotic resistance genes and possibly transfer them to related or unrelated genera within the bacterial community. The extent and molecular mechanisms of MDR development in *H*. *influenzae* are incompletely understood, and more studies are needed.

In this study, we investigated the resistance mechanisms of the MDR *H*. *influenzae* strain Hi-228, expressing phenotypic resistance toward ampicillin, cefotaxime, chloramphenicol, gentamicin, and azithromycin. We identified and characterized a novel ICE, Tn*7100*, containing four resistance-associated transposons with six different resistance genes, including four genes that are previously unreported or rarely reported in *H. influenzae*. To determine the clinical importance of this ICE, we described its genetic structure and environment, ability to transfer, as well as the impact on phenotypic susceptibility, and bacterial fitness.

## Materials and Methods

### Bacterial Isolates

Hi-228 was isolated from a non-hospitalized child with an upper respiratory tract infection in Oslo, Norway, in January 2017. Routine susceptibility testing at the primary laboratory (Fürst) revealed a cefotaxime gradient minimum inhibitory concentration (MIC) of 2 mg/L, and the isolate was sent to the Department of Microbiology, Vestfold Hospital Trust (VHT) for confirmatory testing according to EUCAST recommendations (Leclercq et al., 2013). The isolate was included in an ongoing nationwide study on cefotaxime-resistant *H. influenzae* and characterized with determination of broth microdilution (BMD) MICs and whole-genome sequencing. Multilocus sequence typing (MLST) showed that Hi-228 belonged to ST143. An MDR phenotypic profile was revealed, with matching alterations in PBP3 and six acquired resistance genes (Table 1). As preliminary analyses suggested the presence of a novel ICE, we designed this study to investigate further the molecular basis, transferability, and fitness cost of the acquired resistance mechanisms. All personal data were anonymized before inclusion in this study.

**Table 1:** Characteristics of Hi-228, Hi-122, Hi-142, Hi-143, Hi-147, recipient strain (Rd-Rif), true transconjugants (Tc), and false transconjugants (Tc).

**Strain**	**Broth microdilution (BMD) MIC^a^**						**Gradient MIC^a^**			**MLST**	**Chromosomal resistance**		**Transferable resistance**		
	**CTX**	**AMP**	**CHL**	**AZM**	**GEN**	**RIF**	**AZM**	**GEN**	**RIF**		**PBP3 substitutions (transpeptidase region)**	***rpoB* substitutions**	**Resistance genes^b^**	**ResFinder**	**AMRFinder**	**BLAST**
Hi-228	1	>16	8	>32	>4	≤ 1	>256	128	0.25	ST143	D350N, S357N	None	*bla*_TEM−1B_	AY458016	NG_050145.1	NA
											M377I, S385T		*mef*(E)*^c^*	U83667	NG_047958.1	NA
											L389F, R517H		*mel^c^*	AF274302	NG_048006.1	NA
											T532S, V547I		*catA*2^d^	X53796	NG_047594.1	NA
											Y557H, N569S		*aph*(2″)-Ib^e^	AF337947	NG_047401.1	LR135334
											A586S, S594T		*aac*(6′)-Im	AF337947	NG_047306.1	NA
											A595T, E603D					
Hi-122	1	>16	8	>32	>4	≤ 1	–	–	–	ST143	=Hi-228	None	=Hi-228			
Hi-142	1	>16	>8	>32	>4	≤ 1	–	–	–	ST143	=Hi-228	None	=Hi-228			
Hi-143	1	>16	8	>32	>4	≤ 1	–	–	–	ST143	=Hi-228	None	=Hi-228			
Hi-147	1	>16	8	>32	>4	≤ 1	–	–	–	ST143	=Hi-228	None	=Hi-228			
Rd-Rif	–	–	–	–	–	–	8	2	>32	ST47	None	H526Y	None			
Tc	–	–	–	–	–	–	>256	>1,024	>32	ST47	=Hi-228	H526Y	=Hi-228			
False Tc	–	–	–	–	–	–	>256	≥128	>32	ST143	=Hi-228	D516V/S531F	=Hi-228			

a *MIC, minimum inhibitory concentration (mg/L), CTX, cefotaxime, AMP, ampicillin, CHL, chloramphenicol, AZM, azithromycin, GEN, gentamicin, RIF, rifampicin. Dash, not tested*.

b *All genes 100% coverage and identity unless otherwise noted*.

c *Nomenclature clarifications:*

*mef(E): Reported as mef(A) by ResFinder (U83667) and AMRFinder (NG_047958.1). Nomenclature in this study is according to the original publication of mef(E) (U83667) (Tait-Kamradt et al., 1997) and a minireview by Klaassen and Mouton (Klaassen and Mouton, 2005)*.

*mel: Reported as msr(D) by ResFinder (AF274302) and AMRFinder (NG_048006.1). Nomenclature in this study is according to the original publication of mel and the macrolide efflux genetic assembly (mega) element (AF274302) (Gay and Stephens, 2001)*.

d *catA2: Sequence identity 89.56% with catA (X53796), 100% identity to NG_047594.1, and the catA-like gene in Tn6686 (Hegstad et al., 2020)*.

e *aph(2″)-Ib: Reported as aph(2″)-Ib (AF337947) by ResFinder and aph(2″)-IIa (NG_047401.1) by AMRFinder; both with an identity of 99.89%*.

Four additional ST143 isolates (Hi-122, Hi-142, Hi-143, and Hi-147) with phenotypic and genotypic MDR profiles identical to Hi-228, isolated at the same primary laboratory between June 2016 and January 2017, were included for supplemental phylogenetic analyses to investigate whether the five strains constituted a clonal outbreak.

A rifampicin-resistant spontaneous mutant of *H. influenzae* Rd (CCUG 18800, ST47) was used as recipient strain (Rd-Rif) in conjugation experiments (Hegstad et al., 2020).

Pure cultures were kept frozen at −80°C in Microbank vials (Pro-Laboratory Diagnostics, Canada).

### Study Design

Supplementary Figure S1 gives an overview of the study design.

### Ion Torrent Sequencing

Whole-genome sequencing (Ion Torrent) was performed on all clinical isolates and seven colonies from selective agar plates in conjugation experiments. In brief, colonies were cultured on chocolate agar overnight before they were transferred to 500 μl low-TE buffer. DNA was extracted using Qiasymphony SP, the DSP DNA mini kit, and the blood protocol (Qiagen, Germany). Extracted DNA was quantified using Qubit (Thermo Fisher Scientific, USA). Notably, 200 ng DNA was used for library generation using Library Builder and The Ion Xpress™ Plus Fragment Library Kit for the AB Library Builder™ System (Thermo Fisher Scientific). Preparation of template and chip loading was performed using Ion 510™ & Ion 520™ & Ion 530™ Kit-Chef, and 30 pM was sequenced in the S5™ XL System (Thermo Fisher Scientific).

### PacBio SMRT Sequencing

Hi-228 was cultured on chocolate agar overnight, and a single colony was transferred to brain heart infusion (BHI) broth and grown overnight in a CO_2_ atmosphere. Genomic DNA was extracted using the Promega Wizard Genomic DNA Purification Kit (Promega, USA) and lysozyme (Sigma Aldrich Norway AS). This resulted in a concentration of 250 ng/μl (Qubit) and a 260/280 ratio of 1,862 (GenQuant 1300). Whole-genome sequencing was performed by the Norwegian Sequencing Centre (Oslo, Norway) using the Single-Molecule Real-Time (SMRT) sequencing technology of Pacific Biosciences. The library was prepared using the Pacific Biosciences protocol for Multiplexed Microbial Libraries Using SMRTbell® ExpressTemplate Prep Kit 2.0. DNA was sheared to 12–16 kb fragments using g-tubes from Covaris. Samples were pooled during library prep in (more or less) equimolar. The final library was size-selected using BluePippin with an 8 kb cutoff. The library was sequenced on one 8M SMRT cell on Sequel II instrument using Sequel II Binding kit 2.0 and Sequencing chemistry version 2.0. Loading was performed by diffusion, movie time: 30 h, pre-extension time: 2 h. Of note, 43.5 Gb of library bases were produced. Reads were demultiplexed using Demultiplex Barcodes pipeline using SMRT Tools (SMRT Link version 7.0.0.63985, SMRT Tools version 7.0.0.63823). A minimum barcode score of 26 was used. Reads were assembled using the Microbial Assembly pipeline on SMRT Link (version 9.0.0.92188). Assembly was run using 3 Mb as the expected genome size.

### Bioinformatic Analyses

The overall quality of the raw Ion Torrent reads was examined using AfterQC version 0.9.6 (Chen et al., 2017), prior to as well as after trimming. Quality trimming was performed with Trimmomatic version 0.39 (Bolger et al., 2014), and assembly of trimmed reads was performed using SPAdes version 3.14.1 (Bankevich et al., 2012). The reads were trimmed with a sliding window set to 4, PHRED20, and bases below read length 15 were discarded.

Phylogenetic analysis with conventional and core-genome multi-locus sequence typing (MLST and cgMLST) (Meats et al., 2003) was performed using Ridom SeqSphere+ version 8.0 (Ridom GmbH, Münster, Germany) using the five clinical strains investigated in this study together with a collection of 213 clinical isolates of *H. influenzae* from Norway and Sweden (BioProject PRJEB49398^1^). The analysis was based on 1,782 columns, using the reference sequence *H. influenzae* Rd KW20 (GCA_000027305.1^2^) as seed genome, and the recommended option for the handling of missing values (pairwise ignore)^3^ and MST cluster distance threshold set to 25.

Acquired resistance genes were detected using AMRFinder (Feldgarden et al., 2019) and ResFinder (Bortolaia et al., 2020) with 75% identity/coverage thresholds. A BLAST search was performed for hits with identity below 100%. The presence of a putative ICE was investigated by uploading the assembled contigs to ICEfinder^4^.

To explore whether the resistance genes in Hi-228 were located on an ICE, the assembled contigs were imported into Geneious Prime version 2020.0.1 (Kearse et al., 2012). The six genes of interest (Table 1), as well as the previously described ICE Tn*6686* (MN106411), (Hegstad et al., 2020) were used to create a custom database for annotation of genes (minimum BLAST identity 80%). Contigs with annotations were extracted and sorted. The presence and structure of a tentative ICE were visualized by alignment of the extracted contigs and Tn*6686* (MN106411) with MAUVE version 1.1.1 (Darling et al., 2004). A BLAST search was performed to investigate the origin of two large insertions not present in Tn*6686*. Based on the best match, we added annotations from transposon Tn*6822* in *Streptococcus pneumoniae* (MT489699) and plasmid p49969 in *Citrobacter freundii* (CP070545) to the custom database.

Finally, the presence and structure of a novel ICE (assigned Tn*7100*) were confirmed by annotation of the PacBio sequence with Prokka version 1.14.6 and default parameters with the addition of proteins flag, using the custom database. A comparison of the closed sequence of Tn*7100* with Tn*6686* and ICE*Hin1056* (GenBank accession number MN106411 and AJ627386, respectively) was visualized using Easyfig 2.2.2 (Sullivan et al., 2011).

### Antimicrobial Susceptibility Testing

The BMD MIC was determined according to EUCAST recommendations (EUCAST, 2021), using in-house produced Mueller-Hinton Fastidious (MH-F) broth (Oxoid, Thermo Fisher Scientific) and custom MIC panels (Sensititre NONAG7) (Thermo Fisher Scientific), designed to separate between strains with wild type and acquired resistance. Supplemental testing with gradient diffusion tests (Liofilchem, Italy) on MH-F agar (Oxoid) was performed to determine exact MICs for azithromycin, gentamicin, and rifampicin for Hi-228, Rd-Rif, and colonies from conjugation experiments.

### Agar Plates

We used plates containing Columbia agar base (Becton Dickinson Norway AS) supplemented with 15 mg/L NAD (BioNor Laboratories AS, Norway) and 15 mg/L hemin (Sigma Aldrich Norway AS), as well as chocolate agar plates (Oxoid).

Selective agar plates were Columbia agar plates containing rifampicin (10 mg/L) (rif plates) and rifampicin (10 mg/L) + azithromycin (30 mg/L) (rif+azm plates). The plates were incubated at 35°C, 5% CO_2_ for 24 or 48 h before colony counting.

### Conjugation Experiments

To determine whether Tn*7100* is transferable, mating was performed using the method described by Hegstad et al. (2020). Serial dilutions were plated onto rif plates and rif+azm plates for 48 h to determine the viable counts and number of recipients and transconjugants. Mean values for conjugation frequency (number of transconjugants/number of recipients) and its standard error were calculated. Results were obtained from three biological replicates, initiated on separate days.

### Real-Time PCR and Sequencing of PCR Product

Circularization of Tn*7100* in Hi-228 was analyzed by real-time PCR using primers directed outward from the integrated ICE (Table 2). The PCR product was sequenced to confirm the expected joined right and left end sequences and to determine the correct end of Tn*7100*. The PCR product was purified from agarose gel using the Zymoclean Gel DNA Recovery Kit (Zymo Research, USA) and sequenced at Eurofins Genomics.

**Table 2:** PCR primers and probes used in this study.

	**Primer/probe**	**Sequence (5^′^→3')**	**Product size (bp)**	**Reference**
Circularization of ICE	
Tn*7100* inverse	Forward	GCGTTAGTGGATCGATCGTAG	508	Hegstad et al., 2020
	Reverse	CACGACGGGTTAAAAACTCA		
PCR to confirm transconjugants	
*aac(6′)-*Im	Forward	ACAGATGACCGTGTTCTTGAATTC	143	This study
	Reverse	TGCGTAACCGATAGGAATTGTATC		
	Probe	FAM-ACGATTCGTGAGCATTATACAGAGCAATG-BHQ1		
*mef*(E)	Forward	TAATCACTAGTGCCATCCTGCAA	381	This study
	Reverse	ATAGACTGCAAAGACTGACTATAG		
	Probe	FAM-ATCGCAGCAGCTGGTGCAGTGCTT-BHQ1		
*virB4*	Forward	GAAGAAGCACAGCAGGATGATA	361	This study
	Reverse	CTTAAATCCTTGTCATCCTGGCA		
	Probe	FAM-ATATTAAGCCTATAGGAACTGAGGGAAG-BHQ1		
Rd-Rif (Rd-ORF specific PCR)	Forward	TCTAATTATCGGCGCGATTT	463	Hegstad et al., 2020
	Reverse	TCACATCACGATGGAAGGAA		

To determine whether we had true transconjugants, a representative number of colonies (*n* = 87) from selective agar plates (rif+azm) were analyzed by real-time PCR with the detection of marker genes (Table 2). Colonies were cultured on chocolate agar overnight before they were dissolved and frozen in 500 μl low TE-buffer (Thermo Fisher Scientific). DNA was extracted using QIAsymphony and the “DSP DNA Mini kit” (Qiagen). For all PCR analyses using probes, we utilized the DNA Process Control Kit (Roche Diagnostics, Germany) and the following PCR settings: preincubation for 30 s at 95°C and amplification at 95°C for 5 s and 60°C for 30 s. For PCR analyses without probes, we utilized the LightCycler® FastStart DNA Master^PLUS^ SYBR Green I Kit (Roche Diagnostics, Germany) and the following PCR settings: preincubation at 95°C for 10 min and amplification at 95°C for 10 s, 55°C for 20 s, and 72°C for 30 s, with subsequent melting curve from 65 to 97°C using a heating rate of 0.1°C/s.

### Growth Kinetics

To assess the impact of Tn*7100* on bacterial fitness, the exponential growth rates of transconjugant and recipient were determined. Suspensions of BHI supplemented with 2 mg/L NAD and 10 mg/L hemin (sBHI) with an optical density of 1.0 McFarland were prepared for transconjugant and recipient. A total of 500 μl of the two suspensions were diluted in 499.5 ml sBHI. The bacterial suspensions were shaken at 200 rpm at 37°C. Using a spectrophotometer (GeneQuant 1300, GE Healthcare) growth was measured by absorbance at OD_600nm_ by adding samples of 500 μl bacterial suspensions to spectrophotometer cuvettes (Brand) at given intervals for 810 min (first measurement after 60 min and subsequently every 30 min). Growth rates (*r*) were determined using GrowthRates version 3.0 (Hall et al., 2014). Calculations were based on OD_600_ values in the interval 330 min to 600 min, where growth was observed to be exponential. The fitness of the transconjugant was calculated as relative growth rate = *r*_transconjugant_/*r*_recipient_. Results were obtained from three biological replicates, initiated on separate days.

### Head-to-Head Competition Experiments

To further assess the impact of Tn*7100* on bacterial fitness, head-to-head competition assays were carried out between transconjugant and recipient (Rd-Rif). Notably, 5 μl saline suspension of the recipient (1.0 McFarland) was mixed with equal amounts of transconjugant (1.0 McFarland) in 4.99 ml sBHI. The mixture was incubated at 37°C with continuous shaking (200 rpm). Every 24 h for 3 days, 5 μl of the mixture was diluted 1,000 times in fresh sBHI before re-incubation. The number of colony-forming units (CFUs) of recipients and transconjugants was determined before incubation (N_0_) and at every 24 h (N_24_, N_48_, and N_72_); at each time point, 50 μl of the solution was withdrawn, diluted, and plated on selective agar plates (rif and rif+azm). The selection coefficient was calculated as *s* = *b*/ln(1/*d*) with *b* (=slope) obtained from regressing the natural logarithm of the ratio (CFU_transconjugant_/CFU_recipient_) over time points, and *d* as dilution factor at each transfer (in this study, 1:1,000) (Levin et al., 2000). Relative fitness was calculated as *w* = 1 + *s*, where the fitness of the recipient strain equals 1 (Kloos et al., 2021). For the determination of relative competitive fitness, results were obtained from four biological replicates.

At the end of the experiment, colonies (*n* = 10) from a selective agar plate (rif + azm) were collected for real-time PCR analysis to verify ICE stability.

### Statistical Analyses

Statistical analyses were performed using SPSS version 26. Samples were verified for normality using the Shapiro-Wilk test. One-sample comparisons were performed using student *t*-tests, two-sided. Significance levels are indicated as: *p*-value ^*^ <0.05; ^**^ <0.01; ^***^ <0.001.

### Data Availability

Tn numbers for the novel ICE (Tn*7100*) and transposable elements (Tn*7470* and Tn*7471*) were achieved from the Transposon Registry (Tansirichaiya et al., 2019). The whole-genome sequence of Hi-228 (PacBio) has been deposited in the European Nucleotide Archive (ENA) at EMBL-EBI under accession number OW727395 and BioProject accession number PRJEB52501^5^. Tn*7100*, Tn*7470*, and Tn*7471* have been deposited under the same BioProject and accession numbers OW736085, OW736566, and OW737369, respectively.

### Ethics

All personal data in this study were anonymous. The clinical strains were recruited from an ongoing study approved by the Regional Committees for Medical and Health Research Ethics in Norway (reference number 2018/1558) and the Norwegian Data Protection Services (reference number 232381).

## Results

### Characterization of Five Clinical MDR Strains Comprising a Clonal Outbreak in Oslo, Norway 2016–2017

The five clinical strains (Hi-122, Hi-142, Hi-143, Hi-147, and Hi-228) investigated in this study had the same, previously unreported, MDR profile with resistance toward cefotaxime and high-level resistance to ampicillin, chloramphenicol, gentamicin, and azithromycin (Table 1). Phylogenetic analysis with cgMLST showed that these strains were part of a clonal outbreak in Oslo, Norway in the period June 2016–January 2017 (Supplementary Figure S2). Resistance determinants causative for the MDR profile in these strains were found to be substitutions in PBP3 and the following acquired resistance genes: *bla*_TEM−1B_, *catA2, aac(6*′*)*-Im, *aph(2*″*)*-Ib, *mef* (E), and *mel* (see Table 1 for detailed presentation).

### Characterization of the Novel ICE Tn*7100*, Carrying Four Transposons With Six Genes Conferring Resistance to Beta-Lactams, Phenicols, Aminoglycosides, and Macrolides

Bioinformatic analyses revealed that *bla*_TEM−1B_, *catA2, aac(6*′*)*-Im, *aph(2*″*)*-Ib, *mef* (E), and *mel* in Hi-228 were located on a novel, 73.601-bp ICE, assigned Tn*7100*.

**Figure 1:**
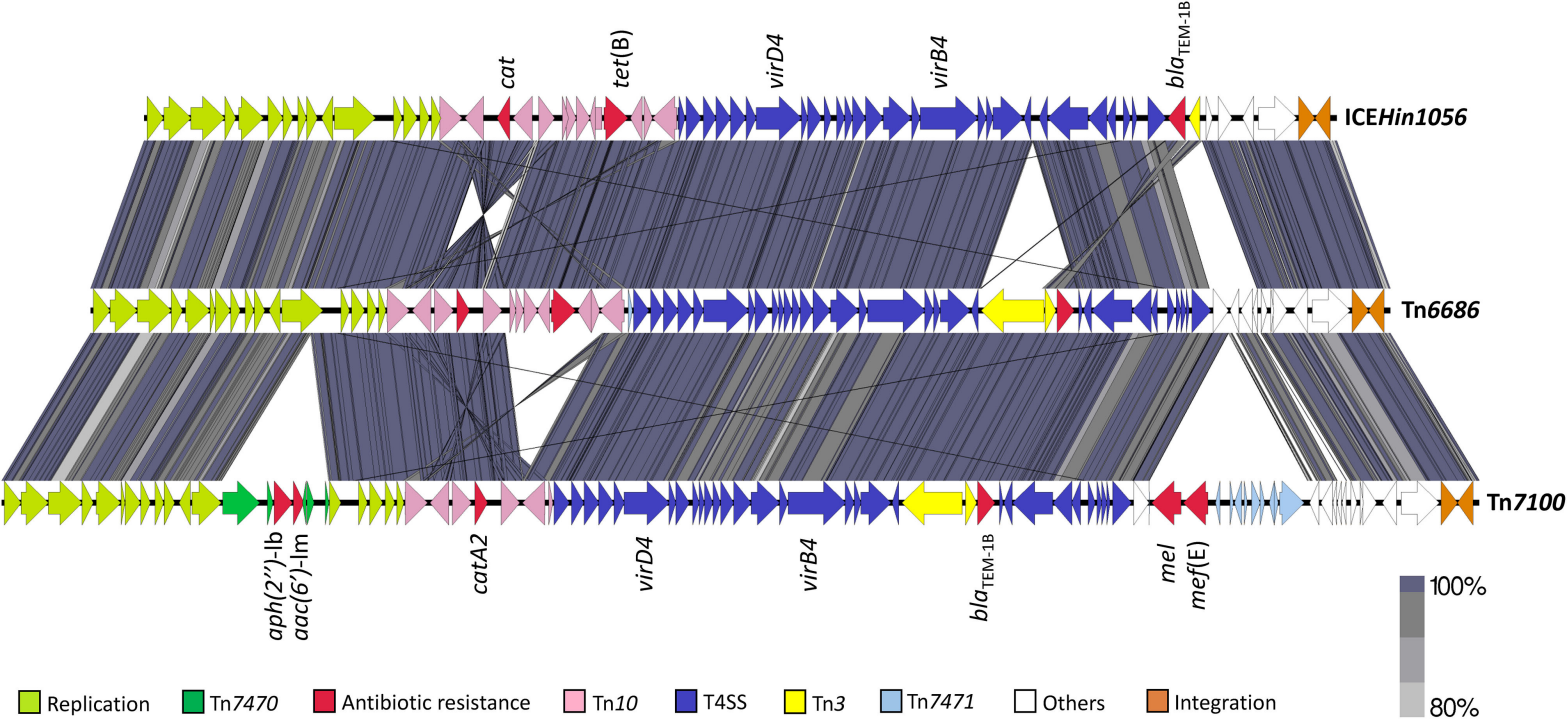
Pairwise comparison of Tn*7100* with Tn*6686* (MN106411) and comparison of Tn*6686* with ICE*Hin1056* (AJ627386). The gray/purple bands represent the forward and reverse matches. Transposable elements, functional regions, and antibiotic resistance determinants are indicated by colored arrows. T4SS, type IV secretion system.

Pairwise BLAST revealed that the overall structure of Tn*7100* was similar to that of Tn*6686*, with *bla*_TEM−1B_ and *catA2* carried by Tn*3* and Tn*10*, respectively (Figure 1) (Hegstad et al., 2020). The major difference was that Tn*7100* lacked *tet*(B) but carried four additional resistance determinants located on two large insertions, representing 7.3 and 10.7% of the total length of Tn*7100*, respectively. The insertions met the criteria for novel transposable elements (Tansirichaiya et al., 2019) and were assigned Tn*7470* (5,380 bp, accession OW736566) and Tn*7471* (7,903 bp, accession OW737369).

**Figure 2:**
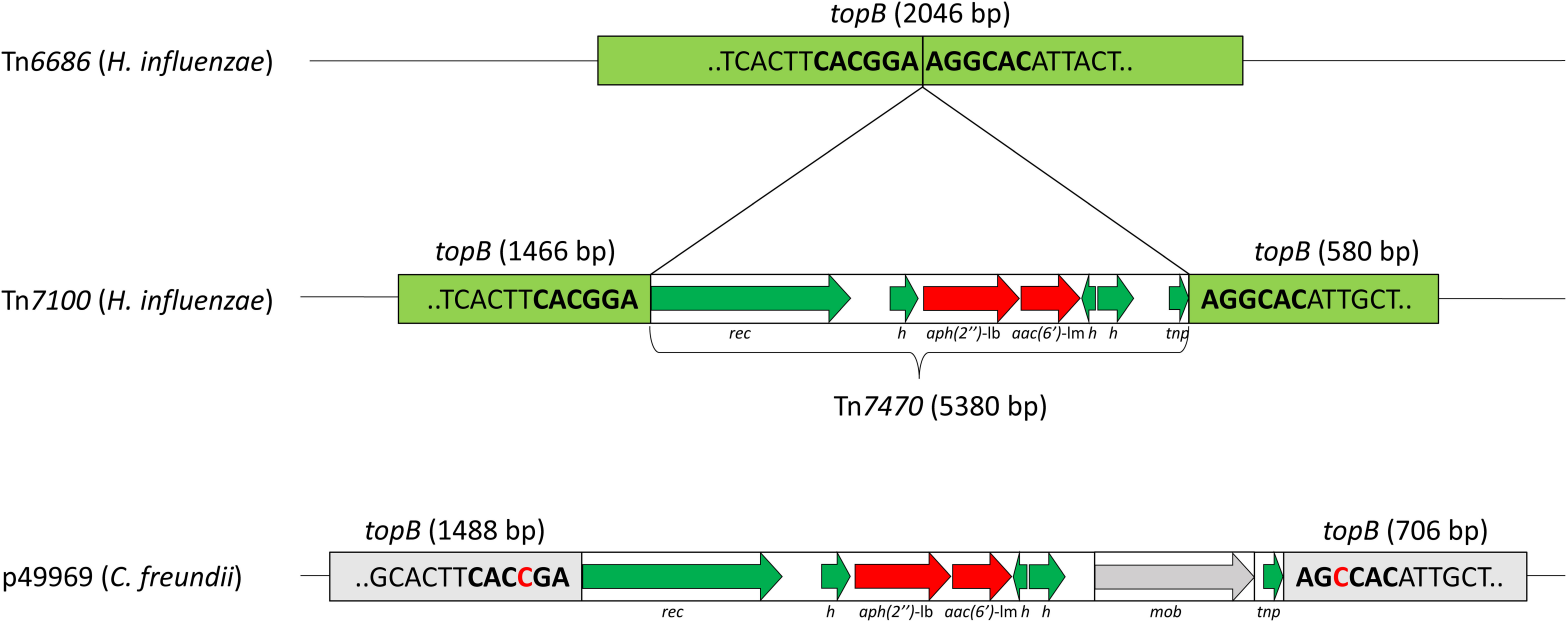
Schematic presentation of the transposable element Tn*7470*, representing 7.3% of Tn*7100*. Refer to Figure 1 for location on Tn*7100*. Tn*7470* consists of a 5,380-bp insertion in the *topB* gene encoding DNA topoisomerase III (light green) (QEA08733), with the insertion site consisting of 6-bp inverted repeats (bold). Tn*7470* carries the aminoglycoside resistance genes *aph(2*″*)-*Ib and *aac(6*′*)*-Im (red arrows) and encodes a recombinase (*rec*) and a TnpW family transposase (*tnp*) and three hypothetical proteins (*h*) (green arrows). Tn*7470* shares 99.96% identity with an insertion in *topB* in plasmid p49969 (CP070545) from *Citrobacter freundii* strain CF49969 but differs from p49969 by lacking a 1,319-bp fragment encoding a MobA/MobL family protein (*mob*). The location of Tn*7470* in topB is similar to p49969 but the target sequences differ by one nucleotide (red text).

The aminoglycoside resistance genes *aac(6*′*)*-Im and *aph(2*″*)*-Ib were located 43 nucleotides apart on Tn*7470* (Figure 2). This transposon was inserted in *topB*, encoding DNA topoisomerase III (accession QEA08733). Tn*7470* also encoded a recombinase and a TnpW family transposase and was flanked by 6-bp inverted repeats with homology to *topB*, typical for transposons (Hallet and Sherratt, 1997). The transposon had a 99.96% identity with a fragment of plasmid p49969 (accession CP070545) in *C. freundii* strain CF49969 (Kraftova et al., 2021), except that one gene upstream of the transposase in p49969, encoding a MobA/MobL family protein, was lacking in Tn7*470*. The location of the transposon in *topB* was similar in Tn*7100* and p49969, with one nucleotide difference in each flanking repeat.

**Figure 3:**
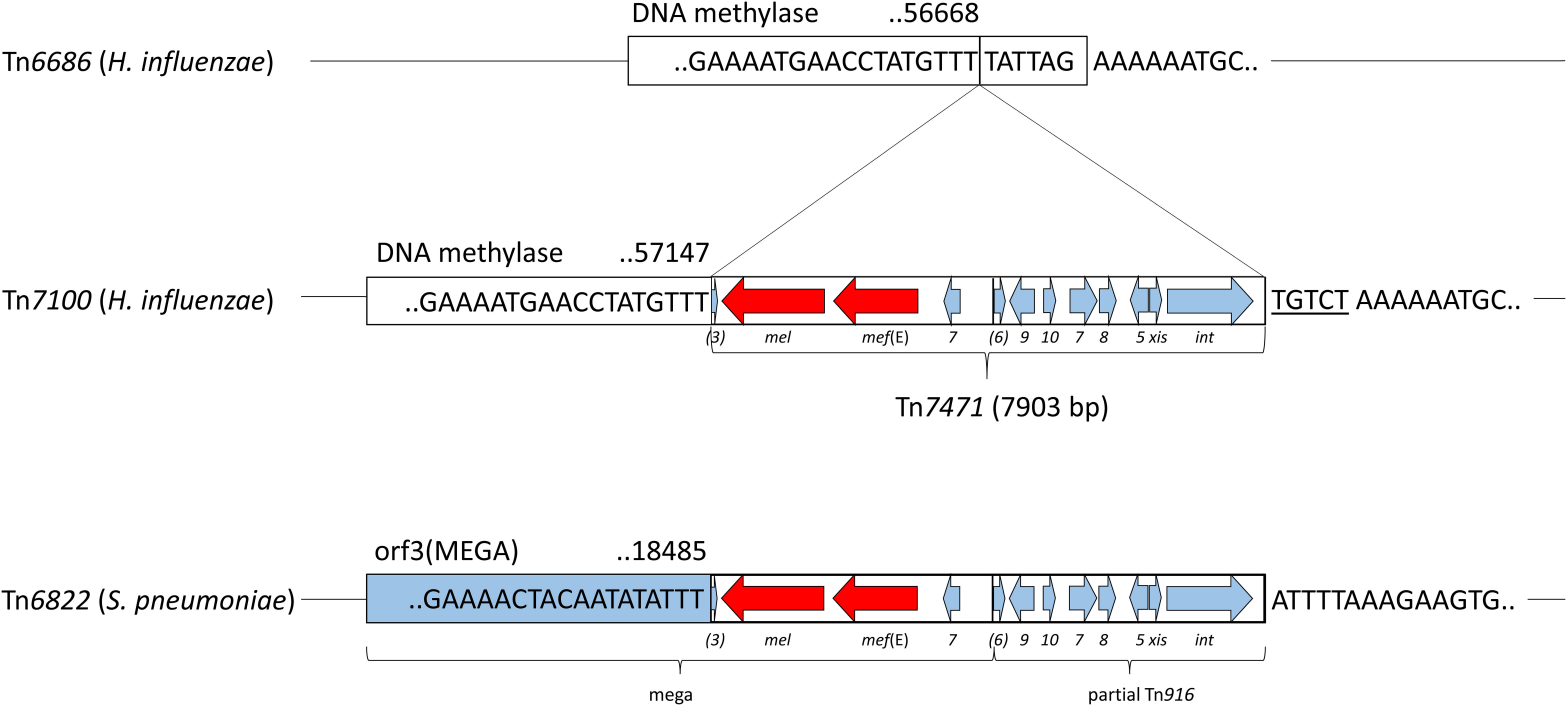
Schematic presentation of the transposable element Tn*7471*, representing 10.7% of Tn*7100*. Refer to Figure 1 for location on Tn*7100*. Tn*7471* consists of a 7,903-bp insertion in the DNA methylase gene (QEA08786) and has a 99.49% identity to a fragment of the conjugative transposon Tn*6822* (MT489699) in *Streptococcus pneumoniae* strain 080217 (NZ_JABAHD010000004, bottom). Top; fragment of Tn*6686* (MN106411) with a vertical line indicating the insertion site, 6 bp within the end of the DNA methylase gene. Tn*7471* contains a truncated macrolide efflux genetic assembly (mega) element (AF274302) with *orf3* (incomplete), macrolide resistance genes *mef* (E) and *mel*, and *orf7* of mega. Furthermore, Tn*7471* contains eight genes of the conjugative transposon Tn*916* (U09422); orfs 5-10 (*orf6* incomplete), and the *xis-*Tn and *int-*Tn genes associated with excision and integration in conjugative transposition. The junction between mega and Tn*916* is indicated by a vertical line. A tentative 5-bp coupling sequence is indicated on the right flank of Tn*7471* (underlined).

The macrolide resistance genes *mel* and *mef* (E) were located adjacently on Tn*7471*, which had 99.49% identity to a fragment of transposon Tn*6822* (accession MT489699) in *S. pneumoniae* strain 080217 (accession NZ_JABAHD010000004) (Nikolaou et al., 2020) (Figure 3). Tn*7471* was inserted 6 bp within the end of the DNA methylase gene (accession QEA08786) on Tn*7100*. The transposon carried a truncated 4,036-bp macrolide efflux genetic assembly (mega) element (accession AF274302) consisting of *mel, mef* (E), and *orf7*, all sharing 100% sequence identity with Tn*6822*. Tn*7471* also contained the terminal 16 bp of *orf3* upstream of *mel*, while *orfs* 4–6 of a complete mega element was lacking (Gay and Stephens, 2001; Del Grosso et al., 2004). Downstream of mega was eight genes of the conjugative transposon Tn*916*, originally found in *Enterococcus faecalis* (accession U09422) (Franke and Clewell, 1981). The eight genes were *orfs* 5-10 (incomplete *orf6*) and *xis*-Tn and *int-*Tn genes, associated with excision and integration in conjugative transposition (Storrs et al., 1991). The mega element was inserted into *orf6* of Tn*916*. The junction between mega and Tn*916* was located 492 bp downstream of *orf7* of mega, where Tn*916* started with nt 26 of *orf6* (Del Grosso et al., 2004).

Almost all sequence differences (38/40) in Tn*7471* compared with Tn*6822* occurred in the terminal 109 bp of *int*-Tn (88.07% identity) or downstream of this gene (85.63% identity). The terminal seven bases in Tn*7471* were identical to Tn*6822* (TTTGTTT), while the downstream coupling sequence, which is transferred with the transposon from the previous host (Rudy and Scott, 1994), differed between Tn*7100* and *S. pneumoniae* strain 080217.

### Conjugative Transfer of Tn*7100* Among *H. influenzae* Strains

**Figure 4:**
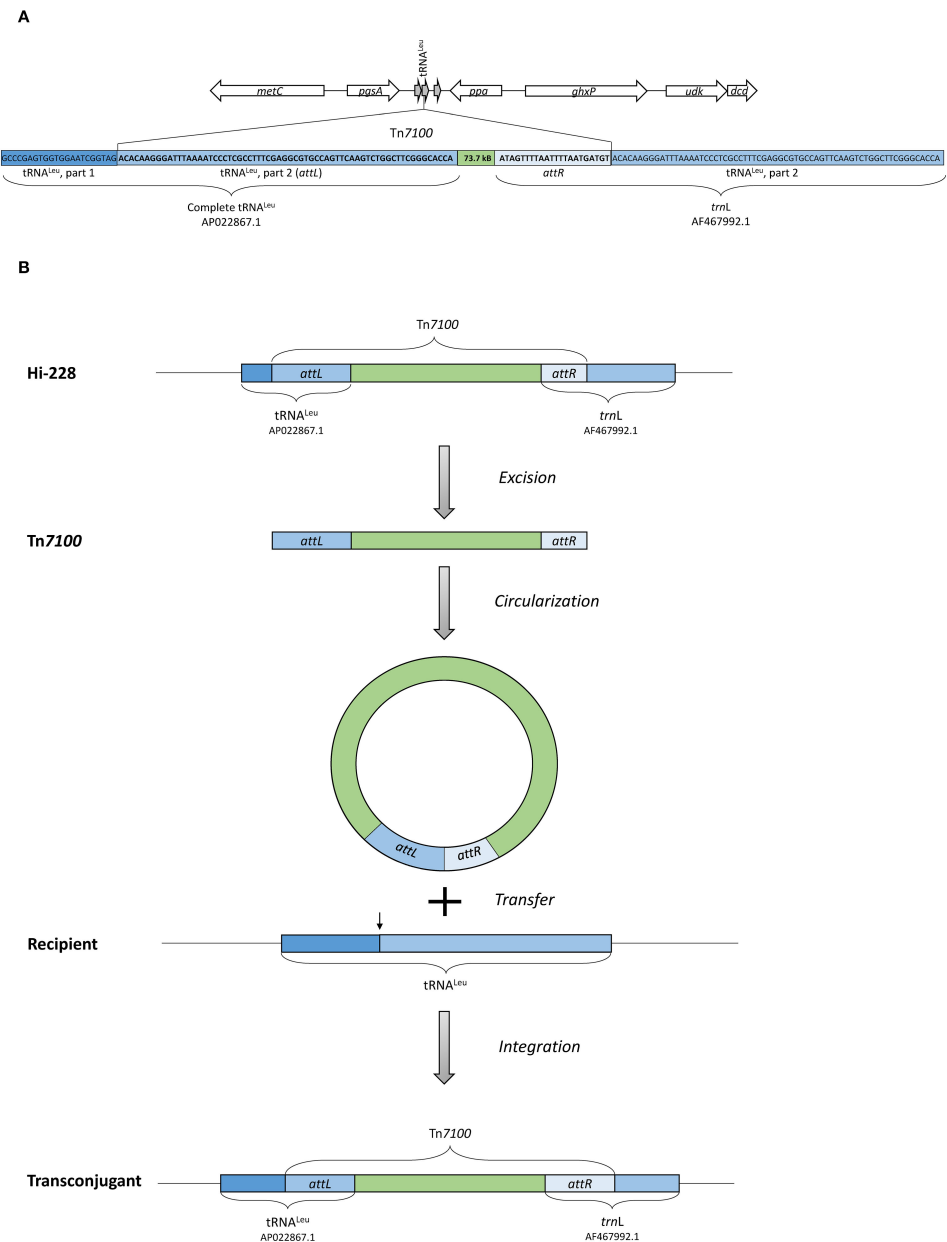
**(A)** Illustration of the insertion point of Tn*7100* in Hi-228, which is located in a tRNA^Leu^ gene. The insertion of Tn*7100* results in a complete tRNA^Leu^ gene (AP022867.1) at the left attachment site (attL) and a complete *trn*L gene (AF467992.1) at the right attachment site (attR). **(B)** Illustration of excision, circularization, transfer, and integration of Tn*7100* from Hi-228 to the recipient strain, as confirmed by experimental procedures in this study.

Sequence analysis demonstrated that the insertion point of Tn*7100* in Hi-228 was located in a tRNA^Leu^ gene, a common insertion site for ICEs in *H. influenzae* (Figure 4A) (Dimopoulou et al., 2002). A circular form of Tn*7100* was demonstrated in Hi-228 using primers directed outward from the integrated ICE (Table 2). Sequencing of the PCR product confirmed the expected joined right and left end sequences and the correct ends of the ICE (data not shown).

To determine the transferability of Tn*7100*, we performed conjugation experiments. The number of colonies on selective agar plates following mating corresponded to transfer frequencies ranging from 5 × 10^−8^ to 4 × 10^−7^ (transconjugants/recipients) (Figure 4B). Preliminary confirmatory analysis with the determination of MIC was performed on 28 colonies from selective agar plates. These colonies were resistant to rifampicin (MIC > 32), azithromycin (MIC > 256), and gentamicin (MIC ≥128).

However, molecular confirmatory analysis using real-time PCR showed that only five out of 87 colonies (6%) on selective agar plates were true transconjugants. The true transconjugants were confirmed by Ion Torrent sequencing, revealing the same MLST profile as Rd-Rif (ST47) (Table 1). Conversely, Ion Torrent sequencing of three suspected false transconjugants (based on PCR results) showed that these colonies had the same MLST profile as Hi-228 (ST143) and had acquired rifampicin resistance-associated substitutions in the *rpoB* gene (D516V or S531F) (Zaw et al., 2018), while plated on selective agar plates (Supplementary Figure S3) (Table 1). These substitutions differed from the substitution in the *rpoB* gene found in the recipient strain Rd-Rif (H526Y).

We found that Tn*7100* was transferable with a conjugation frequency of <5 × 10^−8^. Based on the PCR results showing that only 6% of the colonies on the selective plates were true transconjugants, we estimated a conjugation frequency of ~3 × 10^−9^ (5 × 10^−8^ × 6%).

### Fitness Effects of Tn*7100*

**Figure 5:**
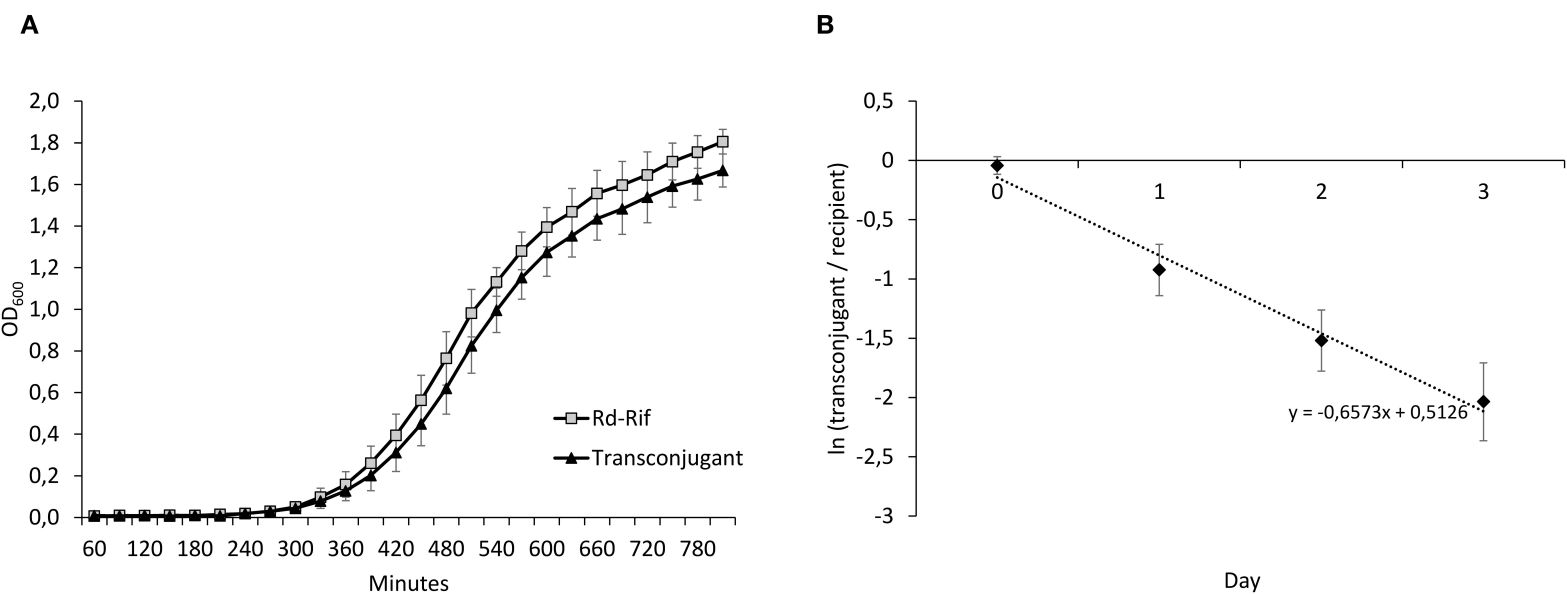
**(A)** Growth curves of Rd-Rif and the transconjugant. **(B)** Results from head-to-head competition experiments between Rd-Rif and the transconjugant, displaying changes in ln(transconjugant/recipient) over time. The average regression (dashed line) is displayed. The negative slope value reflects a fitness cost of Tn*7100*. This was confirmed by calculations of relative fitness (*w*) of the transconjugant (*w* = 0.90, *p* = 0.002). Error bars indicate ± standard deviation.

To determine the fitness effect of Tn*7100* uptake, we measured exponential growth rates of Rd-Rif and a (true) transconjugant (Figure 5A). Relative growth rates (*r*_transconjugant_/*r*_recipient_) showed no significant difference between Rd-Rif and the transconjugant (*p* = 0.812). Head-to-head competition experiments between Rd-Rif and transconjugant showed that the transconjugant had significantly reduced relative fitness compared with Rd-Rif (*w* = 0.90, *p* = 0.002) (Figure 5B). ICE stability throughout the experiment was verified by real-time PCR analysis of colonies from selective agar plates.

## Discussion

The five *H. influenzae* strains investigated in this study were part of a clonal outbreak in Oslo, Norway and expressed resistance toward ampicillin, cefotaxime, chloramphenicol, gentamicin, and azithromycin. To the best of our knowledge, this remarkable MDR profile, caused by a combination of six acquired resistance genes, is previously unreported.

All six resistance genes are carried on a novel ICE, Tn*7100*, with an overall structure quite similar to that of Tn*6686* (Figure 1). Both ICEs contain identical copies of *bla*_TEM−1B_, and the *catA2* gene on Tn*7100* has 100% identity with the “*catA*-like” gene present in Tn*6686* (Hegstad et al., 2020).

In addition, Tn*7100* contains the aminoglycoside resistance genes *aph(2*″*)*-lb and *aac(6*′*)*-lm, located on the novel transposon Tn*7470*. To the best of our knowledge, neither genes have been described in *H. influenzae*. The gene pair, which encodes an aminoglycoside phosphotransferase and acetyltransferase, both mediating aminoglycoside resistance through drug inactivation, appears to be transferred horizontally as a unit (Chow et al., 2001). Notably, Tn*7470* is 99.96% identical to a fragment of the carbapenemase-encoding plasmid p49969 in a Czech strain of *C. freundii*, with only one mismatch in *aac(6*′*)*-lm. This observation suggests that *aph(2*″*)*-lb and *aac(6*′*)*-lm may have been transferred to *H. influenzae* from *Enterobacterales*, but the primary origin of Tn*7470* remains unknown (Kraftova et al., 2021). Notably, *Enterobacterales* frequently colonize the respiratory tract in children and adults with chronic respiratory disease (Soler et al., 1998; Robinson, 2004).

The presence of inverted repeats (IRs) at the insertion site in *topB* suggests that Tn*7470* is integrated into Tn*7100* by site-specific recombination (Hallet and Sherratt, 1997). The region surrounding the insertion site in *topB* is relatively conserved, and the slight sequence variation between Tn*7100* and p49969 is apparently no hindrance to recombination. Importantly, the protein encoded by the intrinsic *topB* in the reference strain *H. influenzae* Rd KW20 has only 42% similarity with the protein encoded by *topB* on Tn*7100*, and neither the IR flanking Tn*7470* on Tn*7100* nor the slightly different IR flanking the corresponding insertion on p49969 are present in the genome of Rd KW20. Acquisition of Tn*7470* is, therefore, unlikely in *H. influenzae* strains lacking a mobile genetic element. Conversely, strains possessing an element encoding IR highly similar to Tn*7100* or p49969 may have an increased probability of acquiring Tn*7470*.

Also located on Tn*7100* are the genes *mef* (E) and *mel*, encoding a dual efflux pump, causing resistance toward macrolides. Transferable macrolide resistance genes have been rarely described in *H. influenzae* (Atkinson et al., 2017). The *mef* (E) and *mel* genes are associated with the macrolide efflux genetic assembly (mega) element (AF274302), first described in pneumococci (Gay and Stephens, 2001; Ambrose et al., 2005). The genes *mef* (E) (U83667) and *mef* (A) (U70055) show 90% identity, while some reports distinguish between the two genes, others simply use the term *mef* (A) for both (Klaassen and Mouton, 2005). Nomenclature in this study is according to the original publication of *mef* (E) (Tait-Kamradt et al., 1997) and a minireview (Klaassen and Mouton, 2005). The *mef* (A) gene was reported in two sequencing-based studies of macrolide-resistant *H. influenzae* from Japan (Seyama et al., 2017; Hara et al., 2019), whereas *mef* (E) has not been reported in this species. Sequence analysis of the *mef* gene on Tn*7100* shows 100% identity to *mef* (E) (U83667), 90% identity to *mef* (A) (U70055), and 99% identity to the gene reported as *mef* (A) by Seyama et al. (LC168847.1) (Seyama et al., 2017). Furthermore, the gene reported as *mef* (A) by Hara et al. (sequence unavailable) (Hara et al., 2019) had a 99% identity with LC168847.1 (Seyama et al., 2017). This indicates that the macrolide resistance genes previously reported as *mef* (A) in *H. influenzae* in fact are identical or highly similar to *mef* (E), also carried by Tn*7100*. This underlines the importance of a clear nomenclature in this field, and the value of carefully curated databases.

In Tn*7100, mef* (E) and *mel* are located on a mega element, inserted into *orf6* of Tn*916* as previously reported in *S. pneumoniae* (Del Grosso et al., 2004). Tn*916* is the first member of the Tn*916*-Tn*1545* family of conjugative transposons originally associated with *tet*(M)-mediated resistance to tetracyclines (Roberts and Mullany, 2011). Notably, the truncated mega element in Tn*7100* appears to have hijacked the transposition machinery of Tn*916* to form the novel, independent transposon Tn*7471*. The transposon shares >99% identity with fragments of transposons described in pneumococci, such as Tn*2010* (Del Grosso et al., 2007) and Tn*6822* (Nikolaou et al., 2020). Tn*916* typically integrates into a target site characterized by a stretch of A's separated from a T-rich region by 5–7 bases (Rudy and Scott, 1994), which is a precise description of the insertion site of Tn*7471* in Tn*7100*. Furthermore, integration involves the transfer of a 5 or 6-bp coupling sequence, located adjacent to the end of the transposon, from the donor to the recipient. Integration of Tn*7471* in Tn*7100* appears to have resulted in the replacement of the terminal six bases of the DNA methylase gene with the 5-bp coupling sequence TGTCT. This sequence is different from the coupling sequence associated with Tn*6822* in *S. pneumoniae* strain 080217, excluding the latter as a donor. A BLAST search using Tn*7471* as input gave the best match (99.52%) with a *Streptococcus oralis* strain from Japan (osk_001, accession AP018338) (Yoshizawa et al., 2018). This strain carries a transposon with a tentative 5- or 6-bp coupling sequence (GTCTA/GTCTAA) identical to the sequence starting one nucleotide downstream from Tn*7471* on Tn*7100*, suggesting that Tn*7471* may originate from oral streptococci. This hypothesis is supported by a recent investigation of Tn*916*-like elements in oral streptococci in Norway, which revealed that strains possessing the transposition machinery of Tn*916* (*xis-*Tn and *int-*Tn) in the absence of *tet*(M) are frequent (Lunde et al., 2021).

In contrast to the truncated mega element on Tn*7100*, complete mega elements harboring *mef* (E) and *mel*, located on *tet*(M)-carrying Tn*916*-like structures, have been reported in *H. influenzae* from Japan (Seyama et al., 2017) and *Haemophilus parainfluenzae* from Switzerland and Spain (Endimiani et al., 2017; Sierra et al., 2021). The mega element on Tn*7100* also has 99% identity with the corresponding fraction of the first mega element found in *Haemophilus parainfluenzae* (KJ545575) (Endimiani et al., 2017). Notably, a BLAST search identified an unreported ICE with a highly similar structure to Tn*7100* (coverage 82%, identity 99.75%) in *H. influenzae* strain BgEED16 isolated in Bangladesh (BioProject PRJEB32184, accession CABFLD010000039). The ICE is located on an 85.569-bp contig and differs from Tn*7100* mainly by lacking Tn*7470* and the Tn*10* segment harboring *catA2*, while the sequence corresponding to Tn*7471* and the adjacent coupling sequence is 100% identical to Tn*7100*. The MLST profile of strain BgEED16 (ST2209) is unrelated to that of Hi-228 (ST143). In summary, this suggests that mega elements containing *mef* and *mel* genes may have been transferred from *S. pneumoniae* or oral streptococci to *H. parainfluenzae* and *H. influenzae* and are now spread globally.

This report highlights the emergence and clonal spread of an *H. influenzae* strain with an unusual MDR profile including transferable, high-level resistance to gentamicin and azithromycin. Although far from first-line therapy in *H. influenzae* infections, both drugs are important for the management of selected patient groups. Gentamicin in combination with ceftriaxone is the recommended treatment of infective endocarditis with *Haemophilus* spp. or other species belonging to the HACEK group (*Aggregatibacter, Cardiobacterium, Eikenella*, and *Kingella*) (Habib et al., 2015). Gentamicin is also used for topical treatment of eye infections. Importantly, as interpretive criteria for antimicrobial susceptibility testing of *Haemophilus* spp. against aminoglycosides are neither established by EUCAST (EUCAST, 2022) nor by the Clinical and Laboratory Standards Institute (CLSI, 2021), treatment is likely to be empiric in many cases, and recognition of acquired high-level resistance may be delayed.

Azithromycin is one of the recommended options for prophylaxis or treatment of respiratory *H. influenzae* infections in patients with underlying diseases, such as cystic fibrosis (Castellani et al., 2018), bronchiectasis (Polverino et al., 2017), COPD (GOLD, 2021), and common variable immunodeficiency (CVID) (Bonilla et al., 2016). Notably, WGS-based detection of resistance genes in paired sputum samples revealed a significant increase in oral streptococci carrying *mef* and *mel* genes during azithromycin therapy (Taylor et al., 2019). These genes are likely carried on Tn*916*-like elements, underlining the potential for inter-species, within-host horizontal spread of macrolide resistance between bacteria sharing the same anatomic niche, as shown in this study. Similar to gentamicin, EUCAST has not defined clinical breakpoints for azithromycin and *H. influenzae* (EUCAST, 2022). Therapy is, therefore, likely to be empiric in most cases, and acquired resistance may thus go unnoticed.

Our results demonstrated that the enzymes involved in excision and circularization of Tn*7100* are functional and that this ICE was transferable to the Rd-derived strain used as the recipient in this study, with a relatively low transfer frequency (~3 × 10^−9^). For comparison, ICE*Hin1056* transfer frequencies range from 10^−1^ to 10^−9^ (Juhas et al., 2007b). It is a weakness of our study that we performed mating experiments with Rd-Rif as the only recipient, and we cannot exclude that using clinical strains of *H. influenzae* as recipients would have affected the observed transfer frequencies.

To assess the fitness effects of Tn*7100*, we conducted growth kinetics and head-to-head competition experiments. Competition experiments offer a more precise measurement of fitness, as they allow comparison throughout several consecutive growth cycles (Lenski, 1991). We found that uptake of Tn*7100* was associated with significantly reduced relative fitness (*p* = 0.002). These results indicate that clinical strains carrying Tn*7100* will be outcompeted in an antibiotic-free environment. Consequently, in the absence of antibiotic selective pressure, survival and further distribution of such strains might be limited even when the horizontal transfer or clonal spread occurs.

To summarize, Tn*7100* is a novel ICE containing four resistance-associated transposons with six different resistance genes, including four genes that are previously unreported or rarely reported in *H. influenzae*. These genes, encoding resistance to aminoglycosides and macrolides, are carried on the novel transposable elements Tn*7470* and Tn*7471*. The integration of two novel transposable elements into an ICE belonging to the ICE*Hin1056* family illustrates that the acquisition of an ICE offers a platform from which *H. influenzae* may acquire new mobile genetic elements containing resistance genes from related or unrelated genera. These results provide important knowledge regarding the role of ICEs in genome dynamics. Although Tn*7100* is transferable and mating experiments generated MDR transconjugants, the ICE has a low transfer frequency and imposes a fitness cost on its recipient. Despite this, strain Hi-228 was capable of clonal spread. The increasingly wide range of transferable resistance genes in the *H. influenzae* supragenome complicates the treatment of infections caused by this pathogen, highlighting the importance of rational antibiotic usage to contain antimicrobial resistance and the emergence of MDR strains.

## Data Availability Statement

The datasets presented in this study can be found in online repositories. The names of the repository/repositories and accession number(s) can be found below: https://www.ebi.ac.uk/ena, PRJEB52501; https://www.ebi.ac.uk/ena, OW727395; https://www.ebi.ac.uk/ena, OW736085; https://www.ebi.ac.uk/ena, OW736566; https://www.ebi.ac.uk/ena, OW737369.

## Author Contributions

HJ, KH, and DS conceived and designed the study. TR supplied the study with clinical isolates and metadata. IA performed Ion Torrent sequencing and real-time PCR. NZ and DS performed bioinformatic analyses. HJ performed antimicrobial susceptibility testing, conjugation experiments, growth kinetics experiments, head-to-head competition experiments, and statistical analysis. HJ and DS wrote the first draft of the manuscript. All authors contributed to the manuscript revision and approved the submitted version.

## Funding

This project received funding from the Vestfold Hospital Trust, the South-Eastern Norway Regional Health Authority (project #2020107), and the Norwegian Surveillance Programme for Antimicrobial Resistance (NORM).

## Conflict of Interest

The authors declare that the research was conducted in the absence of any commercial or financial relationships that could be construed as a potential conflict of interest.

## Publisher's Note

All claims expressed in this article are solely those of the authors and do not necessarily represent those of their affiliated organizations, or those of the publisher, the editors and the reviewers. Any product that may be evaluated in this article, or claim that may be made by its manufacturer, is not guaranteed or endorsed by the publisher.

## Footnotes

^1^Cefotaxime-resistant Haemophilus influenzae (ID 815576)—BioProject—NCBI (nih.gov).

^2^ASM2730v1—Genome—Assembly—NCBI (nih.gov).

^3^Missing Values in MST—Ridom SeqSphere + Documentation.

^4^ http://db-mml.sjtu.edu.cn/ICEfinder/ICEfinder.html

^5^ https://www.ebi.ac.uk/ena/browser/view/PRJEB49398

## References

[B1] Ambrose K. D. Nisbet R. Stephens D. S. (2005). Macrolide efflux in *Streptococcus pneumoniae* is mediated by a dual efflux pump (mel and mef) and is erythromycin inducible. Antimicrob. Agents Chemother. 49, 4203–4209. 10.1128/aac.49.10.4203-4209.2005 16189099 PMC1251515

[B2] Atkinson C. T. Kunde D. A. Tristram S. G. (2017). Expression of acquired macrolide resistance genes in *Haemophilus influenzae*. J. Antimicrob. Chemother. 72, 3298–3301. 10.1093/jac/dkx290 28961896

[B3] Bankevich A. Nurk S. Antipov D. Gurevich A. A. Dvorkin M. Kulikov A. S.. (2012). SPAdes: a new genome assembly algorithm and its applications to single-cell sequencing. J. Comput. Biol. 19, 455–477. 10.1089/cmb.2012.0021 22506599 PMC3342519

[B4] Bolger A. M. Lohse M. Usadel B. (2014). Trimmomatic: a flexible trimmer for Illumina sequence data. Bioinformatics 30, 2114–2120. 10.1093/bioinformatics/btu170 24695404 PMC4103590

[B5] Bonilla F. A. Barlan I. Chapel H. Costa-Carvalho B. T. Cunningham-Rundles C. de la Morena M. T.. (2016). International Consensus Document (ICON): common variable immunodeficiency disorders. J. Allergy Clin. Immunol. Pract. 4, 38–59. 10.1016/j.jaip.2015.07.025 26563668 PMC4869529

[B6] Bortolaia V. Kaas R. S. Ruppe E. Roberts M. C. Schwarz S. Cattoir V.. (2020). ResFinder 4.0 for predictions of phenotypes from genotypes. J. Antimicrob. Chemother. 75, 3491–3500. 10.1093/jac/dkaa345 32780112 PMC7662176

[B7] Botelho J. Schulenburg H. (2020). The Role of Integrative and Conjugative Elements in Antibiotic Resistance Evolution. Trends Microbiol. 29, 8–18. 10.1016/j.tim.2020.05.011 32536522

[B8] Castellani C. Duff A. J. A. Bell S. C. Heijerman H. G. M. Munck A. Ratjen F.. (2018). ECFS best practice guidelines: the 2018 revision. J. Cyst. Fibros. 17, 153–178. 10.1016/j.jcf.2018.02.006 29506920

[B9] Chen S. Huang T. Zhou Y. Han Y. Xu M. Gu J. (2017). AfterQC: automatic filtering, trimming, error removing and quality control for fastq data. BMC Bioinform. 18(Suppl. 3), 80. 10.1186/s12859-017-1469-3 28361673 PMC5374548

[B10] Chow J. W. Kak V. You I. Kao S. J. Petrin J. Clewell D. B.. (2001). Aminoglycoside resistance genes aph(2″)-Ib and aac(6′)-Im detected together in strains of both *Escherichia coli* and *Enterococcus faecium*. Antimicrob. Agents Chemother. 45, 2691–2694. 10.1128/AAC.45.10.2691-2694.2001 11557456 PMC90718

[B11] CLSI (2021). Performance Standards for Antimicrobial Susceptibility Testing, 31st Edn. Available online at: https://clsi.org/ (accessed February 15, 2022).

[B12] Darling A. C. Mau B. Blattner F. R. Perna N. T. (2004). Mauve: multiple alignment of conserved genomic sequence with rearrangements. Genome Res. 14, 1394–1403. 10.1101/gr.2289704 15231754 PMC442156

[B13] Del Grosso M. Northwood J. G. Farrell D. J. Pantosti A. (2007). The macrolide resistance genes erm(B) and mef(E) are carried by Tn2010 in dual-gene *Streptococcus pneumoniae* isolates belonging to clonal complex CC271. Antimicrob. Agents Chemother. 51, 4184–4186. 10.1128/aac.00598-07 17709465 PMC2151421

[B14] Del Grosso M. Scotto d'Abusco A. Iannelli F. Pozzi G. Pantosti A. (2004). Tn2009, a Tn916-like element containing mef(E) in *Streptococcus pneumoniae*. Antimicrob. Agents Chemother. 48, 2037–2042. 10.1128/AAC.48.6.2037-2042.2004 15155196 PMC415626

[B15] Dimopoulou I. D. Russell J. E. Mohd-Zain Z. Herbert R. Crook D. W. (2002). Site-specific recombination with the chromosomal tRNA(Leu) gene by the large conjugative Haemophilus resistance plasmid. Antimicrob. Agents Chemother. 46, 1602–1603. 10.1128/aac.46.5.1602-1603.2002 11959612 PMC127194

[B16] ECDC (2019). Haemophilus influenzae – Annual Epidemiological Report for 2017. Stockholm: ECDC.

[B17] Endimiani A. Allemann A. Wüthrich D. Lupo A. Hilty M. (2017). First report of the macrolide efflux genetic assembly (MEGA) element in Haemophilus parainfluenzae. Int. J. Antimicrob. Agents. 49, 265–266. 10.1016/j.ijantimicag.2016.11.006 27986332

[B18] EUCAST (2021). EUCASTReading Guide for Broth Microdilution, EUCAST. 3.0 Edn. European Society of Clinical Microbiology and Infectious Diseases. Available online at: http://www.eucast.org (accessed November 3, 2021).

[B19] EUCAST (2022). Breakpoint Tables for Interpretation of MICs and Zone Diameters, 12.0 Edn. EUCAST. Available online at: http://www.eucast.org (accessed April 11, 2022).

[B20] Feldgarden M. Brover V. Haft D. H. Prasad A. B. Slotta D. J. Tolstoy I.. (2019). Validating the AMRFinder tool and resistance gene database by using antimicrobial resistance genotype-phenotype correlations in a collection of isolates. Antimicrob. Agents Chemother. 63, e00483–19. 10.1128/aac.00483-19 31427293 PMC6811410

[B21] Franke A. E. Clewell D. B. (1981). Evidence for a chromosome-borne resistance transposon (Tn916) in *Streptococcus faecalis* that is capable of “conjugal” transfer in the absence of a conjugative plasmid. J. Bacteriol. 145, 494–502. 10.1128/jb.145.1.494-502.1981 6257641 PMC217299

[B22] Gay K. Stephens D. S. (2001). Structure and dissemination of a chromosomal insertion element encoding macrolide efflux in *Streptococcus pneumoniae*. J. Infect. Dis. 184, 56–65. 10.1086/321001 11398110

[B23] GOLD (2021). Global Strategy for the Diagnosis, Management, and Prevention of Chronic Obstructive Pulmonary Disease 2022 Report. Available online at: https://goldcopd.org/ (accessed December 1, 2022).

[B24] Habib G. Lancellotti P. Antunes M. J. Bongiorni M. G. Casalta J.-P. Del Zotti F.. (2015). 2015 ESC Guidelines for the management of infective endocarditis: the Task Force for the Management of Infective Endocarditis of the European Society of Cardiology (ESC) Endorsed by: European Association for Cardio-Thoracic Surgery (EACTS), the European Association of Nuclear Medicine (EANM). *Eur. Heart J*. 36, 3075–3128. 10.1093/eurheartj/ehv319 26320109

[B25] Hall B. G. Acar H. Nandipati A. Barlow M. (2014). Growth rates made easy. Mol. Biol. Evol. 31, 232–238. 10.1093/molbev/mst187 24170494

[B26] Hallet B. Sherratt D. J. (1997). Transposition and site-specific recombination: adapting DNA cut-and-paste mechanisms to a variety of genetic rearrangements. FEMS Microbiol. Rev. 21, 157–178. 10.1111/j.1574-6976.1997.tb00349.x 9348666

[B27] Hara N. Wajima T. Seyama S. Tanaka E. Shirai A. Shibata M.. (2019). Isolation of multidrug-resistant *Haemophilus influenzae* harbouring multiple exogenous genes from a patient diagnosed with acute sinusitis. J. Infect. Chemother. 25, 385–387. 10.1016/j.jiac.2018.09.015 30482699

[B28] Hegstad K. Mylvaganam H. Janice J. Josefsen E. Sivertsen A. Skaare D. (2020). Role of horizontal gene transfer in the development of multidrug resistance in *Haemophilus influenzae*. mSphere. 5, e00969–19. 10.1128/mSphere.00969-19 31996416 PMC6992377

[B29] Johnson C. M. Grossman A. D. (2015). Integrative and Conjugative Elements (ICEs): what they do and how they work. Annu. Rev. Genet. 49. 577–601. 10.1146/annurev-genet-112414-055018 26473380 PMC5180612

[B30] Juhas M. Crook D. W. Dimopoulou I. D. Lunter G. Harding R. M. Ferguson D. J.. (2007a). Novel type IV secretion system involved in propagation of genomic islands. J. Bacteriol. 189, 761–771. 10.1128/jb.01327-06 17122343 PMC1797279

[B31] Juhas M. Power P. M. Harding R. M. Ferguson D. J. Dimopoulou I. D. Elamin A. R.. (2007b). Sequence and functional analyses of Haemophilus spp. genomic islands. Genome Biol. 8, R237. 10.1186/gb-2007-8-11-r237 17996041 PMC2258188

[B32] Kearse M. Moir R. Wilson A. Stones-Havas S. Cheung M. Sturrock S.. (2012). Geneious Basic: an integrated and extendable desktop software platform for the organization and analysis of sequence data. Bioinformatics 28, 1647–1649. 10.1093/bioinformatics/bts199 22543367 PMC3371832

[B33] Klaassen C. H. Mouton J. W. (2005). Molecular detection of the macrolide efflux gene: to discriminate or not to discriminate between mef(A) and mef(E). Antimicrob. Agents Chemother. 49, 1271–1278. 10.1128/aac.49.4.1271-1278.2005 15793097 PMC1068581

[B34] Kloos J. Gama J. A. Hegstad J. Samuelsen Ø. Johnsen P. J. (2021). Piggybacking on niche adaptation improves the maintenance of multidrug-resistance plasmids. Mol. Biol. Evol. 38, 3188–3201. 10.1093/molbev/msab091 33760032 PMC8321521

[B35] Kraftova L. Finianos M. Studentova V. Chudejova K. Jakubu V. Zemlickova H.. (2021). Evidence of an epidemic spread of KPC-producing Enterobacterales in Czech hospitals. Sci. Rep. 11, 15732. 10.1038/s41598-021-95285-z 34344951 PMC8333104

[B36] Kuo S. C. Chen P. C. Shiau Y. R. Wang H. Y. Lai J. F. Huang W.. (2014). Levofloxacin-resistant haemophilus influenzae, Taiwan, 2004–2010. Emerging Infect. Dis. 20, 1386–1390. 10.3201/eid2008.140341 25061696 PMC4111205

[B37] Leclercq R. Cantón R. Brown D. F. J. Giske C. G. Heisig P. MacGowan A. P.. (2013). EUCAST expert rules in antimicrobial susceptibility testing. Clin. Microbiol. Infect. 19, 141–160. 10.1111/j.1469-0691.2011.03703.x 22117544

[B38] Lenski R. E.. (1991). “Quantifying fitness and gene stability in microorganisms,” in Assessing Ecological Risks of Biotechnology, ed L. R. Ginzburg (Stoneham, MA: Butterworth-Heinemann), 173–192. 10.1016/b978-0-409-90199-3.50015-2 2009380

[B39] Levin B. R. Perrot V. Walker N. (2000). Compensatory mutations, antibiotic resistance and the population genetics of adaptive evolution in bacteria. Genetics 154, 985–997. 10.1093/genetics/154.3.985 10757748 PMC1460977

[B40] Li X. Du Y. Du P. Dai H. Fang Y. Li Z.. (2016). SXT/R391 integrative and conjugative elements in Proteus species reveal abundant genetic diversity and multidrug resistance. Sci. Rep. 6. 37372. 10.1038/srep37372 27892525 PMC5124997

[B41] Lunde T. M. Hjerde E. Al-Haroni M. (2021). Prevalence, diversity and transferability of the Tn916-Tn1545 family ICE in oral streptococci. J. Oral Microbiol. 13, 1896874. 10.1080/20002297.2021.1896874 33796228 PMC7971310

[B42] Meats E. Feil E. J. Stringer S. Cody A. J. Goldstein R. Kroll J. S.. (2003). Characterization of encapsulated and noncapsulated *Haemophilus influenzae* and determination of phylogenetic relationships by multilocus sequence typing. J. Clin. Microbiol. 41, 1623–1636. 10.1128/JCM.41.4.1623-1636.2003 12682154 PMC153921

[B43] Mohd-Zain Z. Turner S. L. Cerdeño-Tárraga A. M. Lilley A. K. Inzana T. J. Duncan A. J.. (2004). Transferable antibiotic resistance elements in *Haemophilus influenzae* share a common evolutionary origin with a diverse family of syntenic genomic islands. J. Bacteriol. 186, 8114–8122. 10.1128/jb.186.23.8114-8122.2004 15547285 PMC529066

[B44] Nikolaou E. Hubbard A. T. M. Botelho J. Marschall T. A. M. Ferreira D. M. Roberts A. P. (2020). Antibiotic resistance is associated with integrative and conjugative elements and genomic islands in naturally circulating *Streptococcus pneumoniae* isolates from adults in Liverpool, UK. Genes 11, 625. 10.3390/genes11060625 32517221 PMC7348760

[B45] Partridge S. R. Kwong S. M. Firth N. Jensen S. O. (2018). Mobile genetic elements associated with antimicrobial resistance. Clin. Microbiol. Rev. 31, e00088–17. 10.1128/cmr.00088-17 30068738 PMC6148190

[B46] Pfeifer Y. Meisinger I. Brechtel K. Grobner S. (2013). Emergence of a multidrug-resistant *Haemophilus influenzae* strain causing chronic pneumonia in a patient with common variable immunodeficiency. Microb. Drug Resist. 19, 1–5. 10.1089/mdr.2012.0060 23095085

[B47] Polverino E. Goeminne P. C. McDonnell M. J. Aliberti S. Marshall S. E. Loebinger M. R.. (2017). European Respiratory Society guidelines for the management of adult bronchiectasis. Eur. Respirat. J. 50, 1700629. 10.1183/13993003.00629-2017 28889110

[B48] Roberts A. P. Mullany P. (2011). Tn916-like genetic elements: a diverse group of modular mobile elements conferring antibiotic resistance. FEMS Microbiol. Rev. 35, 856–871. 10.1111/j.1574-6976.2011.00283.x 21658082

[B49] Robinson J.. (2004). Colonization and infection of the respiratory tract: What do we know? Paediatr. Child Health 9, 21–24. 10.1093/pch/9.1.21 19654976 PMC2719511

[B50] Rudy C. K. Scott J. R. (1994). Length of the coupling sequence of Tn916. J. Bacteriol. 176, 3386–3388. 10.1128/jb.176.11.3386-3388.1994 8195096 PMC205512

[B51] Seyama S. Wajima T. Suzuki M. Ushio M. Fujii T. Noguchi N. (2017). Emergence and molecular characterization of *Haemophilus influenzae* harbouring mef(A). *J. Antimicrob. Chemother*. 72, 948–949. 10.1093/jac/dkw501 27999037

[B52] Sierra Y. González-Díaz A. Carrera-Salinas A. Berbel D. Vázquez-Sánchez D. A. Tubau F.. (2021). Genome-wide analysis of urogenital and respiratory multidrug-resistant *Haemophilus parainfluenzae*. J. Antimicrob. Chemother. 76, 1741–1751. 10.1093/jac/dkab109 33792695

[B53] Skaare D. Anthonisen I. L. Kahlmeter G. Matuschek E. Natas O. B. Steinbakk M.. (2014). Emergence of clonally related multidrug resistant *Haemophilus influenzae* with penicillin-binding protein 3-mediated resistance to extended-spectrum cephalosporins, Norway, 2006 to 2013. Euro Surveill. 19, 10.2807/1560-7917.es2014.19.49.20986 25523969

[B54] Soler N. Torres A. Ewig S. Gonzalez J. Celis R. El-Ebiary M.. (1998). Bronchial microbial patterns in severe exacerbations of chronic obstructive pulmonary disease (COPD) requiring mechanical ventilation. Am. J. Respir. Crit. Care Med. 157(5 Pt 1), 1498–1505. 10.1164/ajrccm.157.5.9711044 9603129

[B55] Storrs M. J. Poyart-Salmeron C. Trieu-Cuot P. Courvalin P. (1991). Conjugative transposition of Tn916 requires the excisive and integrative activities of the transposon-encoded integrase. J. Bacteriol. 173, 4347–4352. 10.1128/jb.173.14.4347-4352.1991 1648556 PMC208095

[B56] Sullivan M. J. Petty N. K. Beatson S. A. (2011). Easyfig: a genome comparison visualizer. Bioinformatics. 27, 1009–1010. 10.1093/bioinformatics/btr039 21278367 PMC3065679

[B57] Tait-Kamradt A. Clancy J. Cronan M. Dib-Hajj F. Wondrack L. Yuan W.. (1997). mefE is necessary for the erythromycin-resistant M phenotype in *Streptococcus pneumoniae*. Antimicrob. Agents Chemother. 41, 2251–2255. 10.1128/aac.41.10.2251 9333056 PMC164101

[B58] Tansirichaiya S. Rahman M. A. Roberts A. P. (2019). The transposon registry. Mob. DNA 10,40. 10.1186/s13100-019-0182-3 31624505 PMC6785933

[B59] Taylor S. L. Leong L. E. X. Mobegi F. M. Choo J. M. Wesselingh S. Yang I. A.. (2019). Long-term azithromycin reduces *Haemophilus influenzae* and increases antibiotic resistance in severe asthma. Am. J. Respir. Crit. Care Med. 200, 309–317. 10.1164/rccm.201809-1739OC 30875247

[B60] Van Eldere J. Slack M. P. Ladhani S. Cripps A. W. (2014). Non-typeable *Haemophilus influenzae*, an under-recognised pathogen. Lancet Infect. Dis. 14, 1281–1292. 10.1016/s1473-3099(14)70734-0 25012226

[B61] WHO (2017). Global Priority List of Antibiotic-resistant Bacteria to Guide Research, Discovery, and Development of New Antibiotics. Geneva: WHO.

[B62] Wozniak R. A. Waldor M. K. (2010). Integrative and conjugative elements: mosaic mobile genetic elements enabling dynamic lateral gene flow. Nat. Rev. Microbiol. 8, 552–563. 10.1038/nrmicro2382 20601965

[B63] Yoshizawa H. Motooka D. Matsumoto Y. Katada R. Nakamura S. Morii E.. (2018). A case of severe soft tissue infection due to *Streptococcus tigurinus* diagnosed by necropsy in which genomic analysis was useful for clarifying its pathogenicity. Pathol. Int. 68, 301–306. 10.1111/pin.12656 29570912

[B64] Zaw M. T. Emran N. A. Lin Z. (2018). Mutations inside rifampicin-resistance determining region of rpoB gene associated with rifampicin-resistance in *Mycobacterium tuberculosis*. J. Infect. Public Health. 11, 605–610. 10.1016/j.jiph.2018.04.005 29706316

