# Supplementary material for: Characterization and Fitness Cost of Tn7100, a Novel Integrative and Conjugative Element Conferring Multidrug Resistance in Haemophilus influenzae
Source: Front Microbiol. 2022 Jul 22;13:945411. doi: 10.3389/fmicb.2022.945411 (PMC9355037; doi:10.3389/fmicb.2022.945411)
Supplement: Supplementary file 1 [file Data_Sheet_1.pdf]

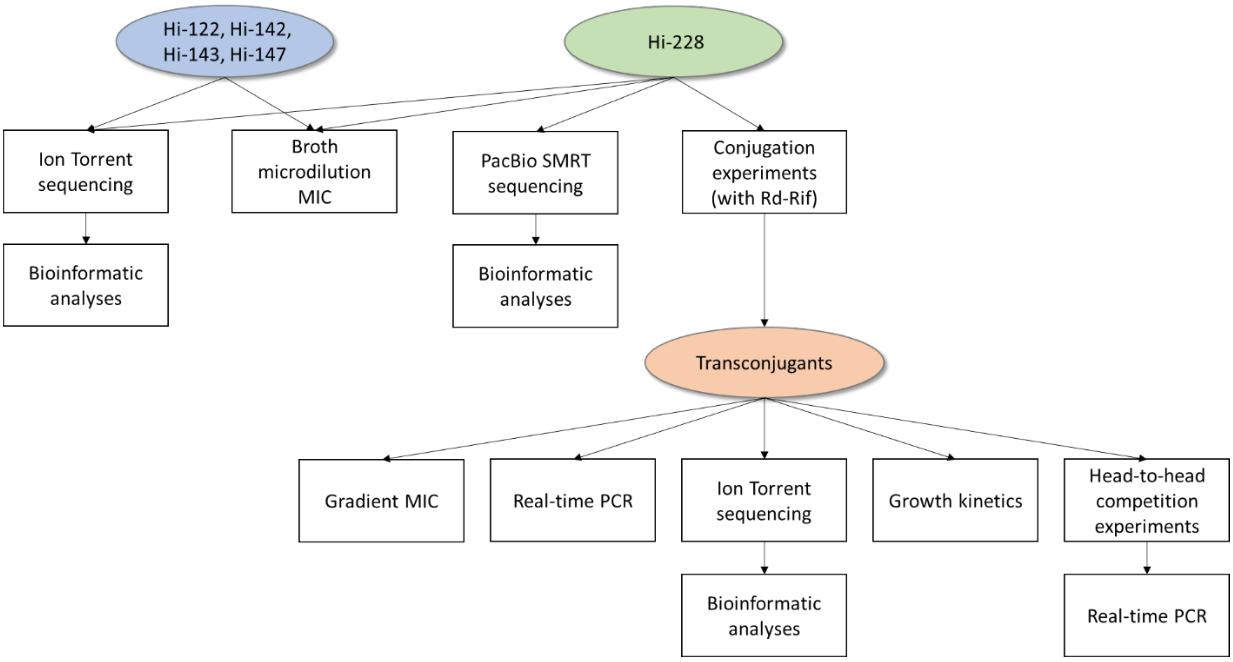

Figure S1: Schematic illustration of the study design and experimental procedures performed on the clinical strains (Hi-122, Hi-142, Hi-143, Hi-147, and Hi-228), recipient strain (Rd-Rif), and transconjugants.

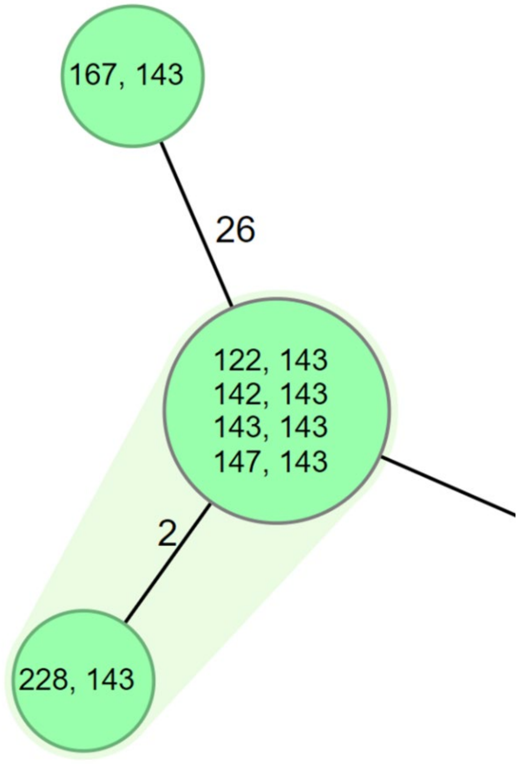

Figure S2: Section of Minimum Spanning Tree (MST) showing results from conventional and core-genome multi-locus sequence typing (MLST and cgMLST) of the five strains investigated in this study together with a collection of 213 clinical isolates of *H. influenzae* from Norway and Sweden (BioProject PRJEB49398[1]). MST cluster distance threshold = 25. Strains Hi-122, Hi-142, Hi-143, Hi-147, Hi-228 belonged to the same MST cluster and were epidemiologically related (Oslo, Norway, June 2016 – January 2017). The closest related strain (Hi-167) was collected in a different geographical area (Trøndelag county, Norway, July 2017) and lacked transferable resistance genes. The closest related ST was ST183 (561 allelic differences). Numbers within circles indicate ID and ST; numbers between circles indicate allelic differences.

[1] Cefotaxime-resistant Haemophilus influenzae (ID 815576) - BioProject - NCBI (nih.gov)

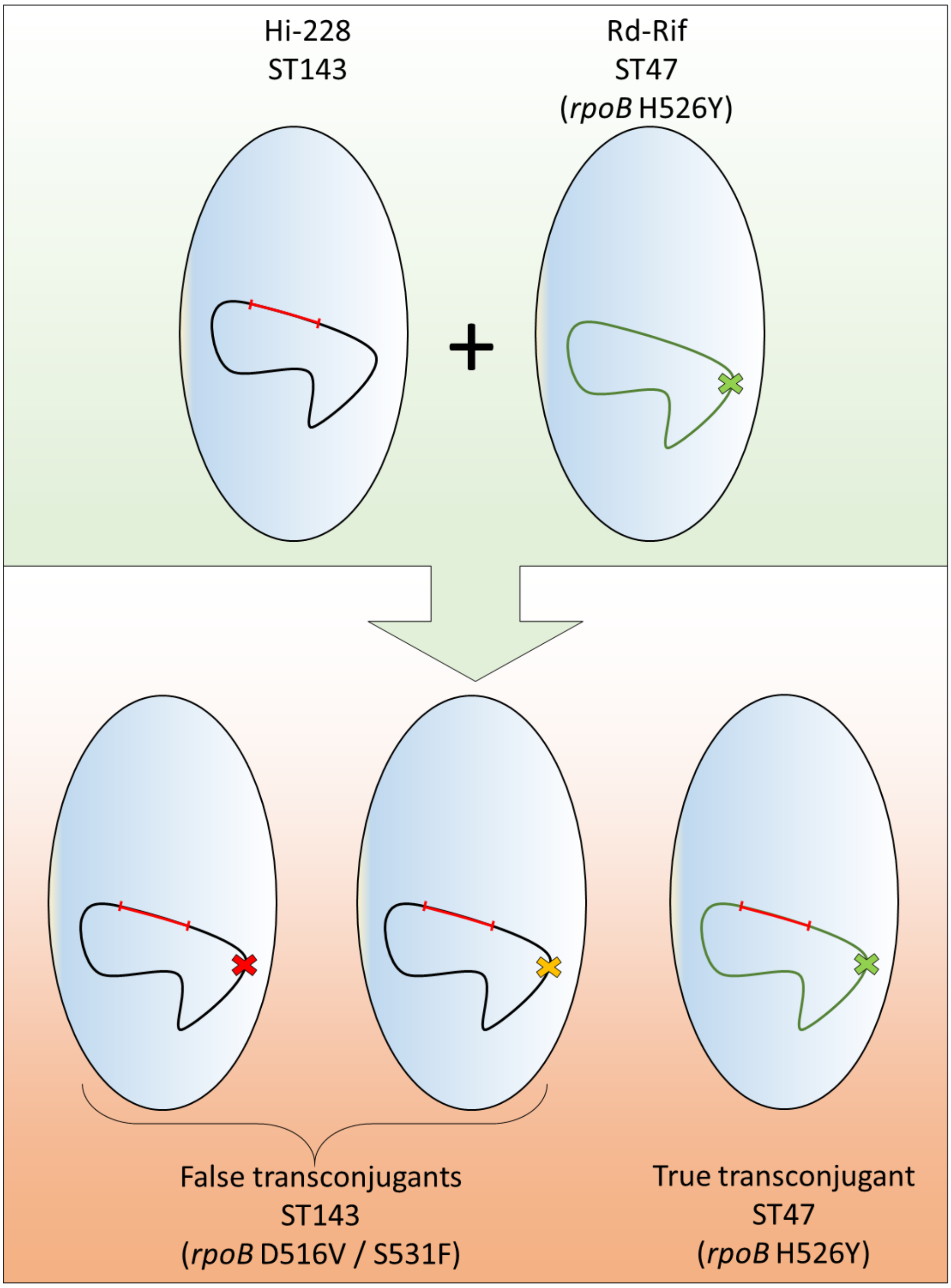

Figure S3: Illustration of the conjugation experiments performed in this study, resulting in both false and true transconjugants. Top: donor (Hi-228) and recipient (Rd-Rif), bottom: false and true transconjugants. When plated on selective agar plates (rif+azm plates), a relatively high proportion of Hi-228 colonies acquired rifampicin resistance-associated substitutions in the *rpoB* gene, resulting in false transconjugants. Tn*7100* indicated in red, donor genome indicated in black, recipient genome indicated in green, substitutions in the *rpoB* gene indicated by colored crosses and described in the figure.
